# The role of contact system in septic shock: the next target? An overview of the current evidence

**DOI:** 10.1186/s40560-017-0228-x

**Published:** 2017-05-30

**Authors:** Henrique Nicola

**Affiliations:** Intensive Care Registrar Trainee, Royal Perth Hospital, 197 Wellington St Perth, Western Australia, 6000 Australia

**Keywords:** Septic shock, Bradykinin, Contact system, Intensive care, Infectious diseases

## Abstract

**Background:**

Septic shock remains challenging to intensive care units worldwide, despite recent documented improvement in mortality over the years. Multiple new therapies have been attempted without success in large clinical trials. Evidence concerning the role of the contact system and bradykinin on septic shock physiological manifestations is shown by this article.

**Objectives:**

The objective of the study is to review the current evidence linking contact system activation and septic shock, as well as efficacy of available therapies targeting this pathophysiological pathway and to evaluate the potential of further researching the matter.

**Results:**

Multiple animal studies are already available and suggestive of a meaningful role of contact system activation on septic shock. However, human trials are still scarce, and the ones available are not enough to establish such a strong connection. Furthermore, attempted therapies have been successful across multiple species, but not as much in humans. Therefore, contact system and septic shock relationship remains plentiful in questions to be answered in the coming years or decades.

**Conclusions:**

Whether the contact system is not as relevant in humans as it is in animals or there is only lack of evidence remains to be explained. The subject is an attractive open field for further research aiming to aid in tackling such a burdensome condition.

## Background

Septic shock remains a major challenge for intensive care units (ICU) worldwide. Although mortality seems to have improved over the last decade [1], there has been no breakthrough or single intervention that allowed for a significant improvement in outcome. Drugs such as activated alpha-drotrecogin, levosimendan, and hydrocortisone failed, as of today, to be a stepping stone in septic shock patient end points [2–4]. Little data, however, has been produced looking at the contact system in this context.

**Table 1:** ᅟ

Author, year of publication	Subject studied	Intervention	Main outcomes analyzed	Results	Reported drug adverse effects
Pixley et al. 1993 [13]	Baboons injected with *Escherichia coli*	C6B7, an antibody to FXII	Mortality, reversible hypotension, and DIC	Null hypothesis rejected for mortality and reversible hypotension, but not DIC	None reported
Ridings et al, 1995 [14]	Porcine models injected with *Pseudomonas aeruginosa*	NPC 17731, a B2R antagonist	SAP, SVRI, CI arterial pH	Significant improvement in SAP, SVRI, and artery pH comparing to septic control, but not in CI	None reported
Shin et al, 1996 [15]	Rats injected with *Pseudomonas elastase*	Soybean trypsin inhibitor (a kallikrein inhibitor)	Hypotension	Hypotension response completely abolished in treatment group	None reported
Fein et al. 1997 [16]	Humans with sepsis and evidence of dysfuntion of two or more organs	Deltaband, a B2R antagonist	Mortality and APACHE III score	No difference in overall mortality or APACHE scores, but significant improvement among gram-negative sepsis patients	No increase in adverse effects compared to placebo. No other safety concern noted
Caliezi et al. 2002 [19]	Humans with severe sepsis or septic shock	C1-inhibitor	Mortality and organ dysfunction	No improvement in mortality, but significant attenuation in the degree of renal dysfunction	None reported
Barratt-Due et al. 2011[20]	Porcine models injected with *Neisseria meningitidis*	Icatiband, aB2R antagonist	Mortality and physiological parameters	No significant difference between the studied groups	None reported
Murugesan et al. 2016 [21]	Rats with polymicrobial severe sepsis (induced by cecal ligation and puncture)	BI113823, a B1R antagonist	Mortality and physiological parameters	Significant improvement in overall mortality and many physiological parameters	None reported

Summary of studies which used pharmacological interventions targeting the contact system and their results. [*FXII* Factor XII, *DIC* disseminated intravascular coagulation, *SAP* systemic arterial pressure, *SVRI* systemic vascular resistance index, *CI* cardiac index, *B2R* bradykinin-2-receptor, *B1R* bradykinin-1-receptor]

## The contact system

**Fig. 1:**
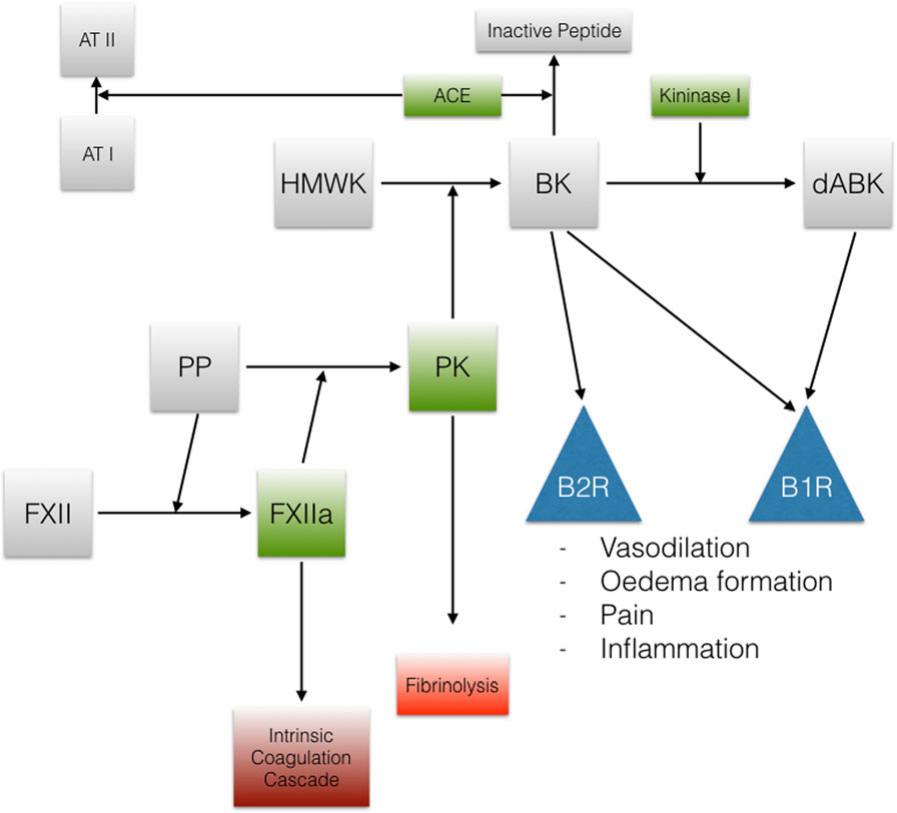
Illustrates the contact system from protein and proteinase interactions until the final action of bradykinin on its receptors, as well as the mechanism by which it influences the coagulation cascade. Note not only the bradykinin but also its product after being transformed by kininase are agonists at the bradykinin-1-receptor. Angiotensin converting enzyme (ACE) also influences the system by being the bradykinin inactivating substance. Please refer to the text for further details. [*FXII* factor XII, *FXIIa* activated factor XII, *PP* plasma prekallikrein, *PK* plasma kallikrein, *HMWK* high molecular weight kininogen, *BK* bradykinin, *dABK* desArg9-bradykinin, *ACE* angiotensin converting enzyme, *AT I* angiotensin I, *AT II* angiotensin II, *B1R* bradykinin-1-receptor, *B2R* bradykinin-2-receptor]

The contact system consists of four plasma proteins and proteinases: coagulation factors XI and XII, plasma prekallikrein (PP), and high molecular weight kininogen (HMWK). These substances normally circulate in the bloodstream or are bound to cell surfaces such as polymorphonuclear neutrophils, endothelial cells, and platelets. When activated, their products participate in numerous disease states, taking an important role in the body’s defense against injuries, having effects on both coagulation and inflammatory response. Its activation relies upon the presence of negatively charged surfaces, and the interaction of coagulation factor XII (FXII) with these lead to its auto activation. Activated FXII allows for PP to be transformed in kallikrein, which exerts two main functions: (1) increases the activity of activated FXII, leading ultimately to the activation of coagulation factor XI (FXI) and the subsequent intrinsic coagulation cascade, and (2) interacts with HMWK, which releases bradykinin, a well-known 9 amino acid peptide with potent pro-inflammatory and vasodilator properties via its G-protein-coupled receptors [5] [Fig. 1].

Bradykinin’s excess has been well linked to hereditary angioedema (caused by C1-inhibitor deficiency, an inhibitor of the complement and contact systems), and drugs that aim for regulation of the contact system are currently mainstay on this condition’s therapy [6]. Activation of contact system has also been associated with inflammatory bowel disease and rheumatoid arthritis, although the relevance of it in human disease is yet to be better determined [7, 8]. In septic shock, its pathophysiological importance is undeniable, however still to be precisely quantified. Relying on the above described mechanisms, it can be hypothesized the contact system has contributory role to the vasodilatory state and capillary leakiness, as well as coagulation derangements commonly present in this condition. Evidence of this relation is summarized over the next section.

## Hereditary angioedema

To better comprehend the contact system and development of therapies aiming to blunt its activity, understanding hereditary angioedema is necessary. Even though hereditary angioedema has little correlation with sepsis and lacks the overwhelming inflammatory response of the latter, researching its pathophysiology and the development of therapeutic options have already provided advances specifically related to the contact system [9]. This condition is caused by the lack of C1-inhibitor, a member of the serpine family of protease inhibitors, which possesses inhibitory activity towards the complement and contact systems. Bradykinin’s role in hereditary angioedema manifestations, such as vasodilation and capillary leakiness leading to oedema in multiple tissues and organs, has been well established. Since that, three classes of medication have been developed to treat the condition: C1 inhibitors, kallikrein inhibitors, and selective bradykinin-2-receptor antagonists, with different degrees of efficacy demonstrated by clinical trials [9].

## Severe infection and contact activation

Sepsis has been newly redefined as “a life-threatening organ dysfunction caused by a dysregulated host response to infection,” whereas septic shock is now “a subset of sepsis in which underlying circulatory and cellular/metabolic abnormalities are profound enough to substantially increase mortality.” This means that the inflammatory response to the offending organism is overwhelming, becoming deleterious. Its pathophysiology involves activation of macrophage by bacterial products and the release of tumor necrosis factor (TNF), along with inflammatory cytokine such as interleukins (IL) 1, 2, 6, 8, and 12, interferon, and platelet activation factor. Further recruitment of defense cells and the release of a cascade of cytokines leads to over-activation of the entire inflammatory cascade and complement system, which, along with activation of the contact system, results in a potentially dramatic clinical picture, characterized by excessive vasodilation, and capillary leakiness. Cardiac depression also occurs commonly, attributed to catecholamine-induced cardiomyocyte toxicity, cytokine-mediated (TNF and IL-1), and mitochondrial dysfunction. Endothelial damage caused mainly by cytokines causes release of tissue factor and activation of the coagulation cascades. As this process is not well controlled, intravascular microthrombi formation and consumption-related coagulopathy results in disseminated intravascular coagulation (DIC) [10]. The following paragraphs explore currently availale evidencelinking septic shock to activation of contact system, and the trials involving pharmacological intervention to this pathophysiological route are summarised in Table 1.

Pixley and colleagues published three studies in 1991, 1992, and 1993 in which they first developed a test by which they used enzyme-linked immunosorbent assay (ELISA) to measure α2-macroglobulin-Kallikrein (α2M-kal) complex levels in the plasma as an indicator of the contact system activation [11]. The following year, they used baboons and models to inject *Escherichia coli* (*E. coli*) in lethal and non-lethal quantities. When measuring levels of HMWK and α2M-kal, the differences between groups were significant, suggesting irreversible hypotension to be related to contact system activation [12]. In the latter study, again using *E. coli* in baboon models, an antibody to FXII (called C6B7) was used to inhibit contact system activation, and hypotension was reversed successfully in the treated group. However, DIC was not different, being present in both treated and untreated groups [13].

Only 2 years later, Ridings and colleagues used porcine models and injected live *Pseudomonas aeruginosa* as well as a bradykinin antagonist (called NPC17731), forming three study arms—control (saline only), septic shock (bacteria only), and treated (bacteria and therapy) groups. Their results corroborate Pixley research, suggesting a significant role of bradykinin in the pathogenesis of septic shock hypotension. NPC17731 successfully reversed hypotension in the group which received the drug [14].

In 1996, Shin et al. hypothesized that reducing bradykinin release in rats septic shock would improve their survival. To demonstrate this effect, their team injected *Pseudomonas elastase* in healthy animals, which increased bradykinin plasma levels from <1 ng/mL to an average of 25 ng/mL and decreased the mean arterial pressure (MAP) by 45 mmHg. They then used the same method, this time after injecting the animals with soybean trypsin inhibitor (SBTI), both native and suc-gel (longer acting), which exert strong inhibition against plasma kallikrein. The results, published by the *Immunopharmacology Journal*, demonstrated no raise in the bradykinin levels and only minimal (<10 mmHg) average fall in MAP in the second set of animals (post SBTI administration), completely abolishing the hypotensive response to the bacteria protease [15].

Fein et al., in 1997 [16], published the first and only human randomized double-blind controlled trial comparing 3 different doses of the bradykinin-2-receptor antagonist deltibant with placebo. It included 504 patients across 47 US hospitals with systemic inflammatory response syndrome (SIRS) and documented evidence of infection, plus either hypotension or evidence of dysfunction of 2 or more organs. The study primary end point was overall 28-day mortality, being their secondary end point organ failure severity scores and subgroup survival by different types of infection. Deltiband failed to reduce the overall 28-day mortality and organ failure scores for any of its tested doses. However, the drug reduced mortality with statistical significance among patients with purely gram-negative infection after severity adjustment (*p* < 0.005). Neither patients with gram-positive nor the ones with mixed infections had reduction in mortality. The cause of this difference of response was called “unclear” by the authors, which than bring 2 hypotheses to explain it: (1) differential mediator response to different classes of organisms and (2) an interaction between severity and drug effect, since patients with gram-negative infections tend as group to be sicker [16].

A brief report, published in 2000, demonstrated retrospectively the finding of isolated prolonged activated partial thromboplastin time (APTT) in seven patients with streptococcal toxic shock syndrome, all normalized after recovery. The authors argue that this finding is a possible surrogate of contact system activation and that it would explain many features of the syndrome [17].

Eight years later, researchers developed an assay measuring the 47 kD light chain of high molecular weight kininogen (47 kD HK), one of the products of cleaving the 120 kD HMWK, which possesses long half-life and is technically less laborious and subject to errors than measuring bradykinin directly. They measured 47 kD HK in six patients with sepsis on days 1–3 and 6–8 of admission and found correlation of the levels measured with severity of disease. Furthermore, they found that 47 kD levels increased early in the course of sepsis [18].

Caliezi et al. was the first group to test the use of C1-inhibitor in severe sepsis and septic shock patients, publishing a small randomized controlled trial, which included 40 patients and comparing the drug with placebo. There was no difference in mortality, but the drug attenuated significantly the degree of renal impairment without producing side effects. Local inhibition of complement and contact activation or prevention of extensive capillary leakage are the two explanations given by the authors to the benefical effect in renal impairment [19].

In 2011, Barratt-Due et al. published a study evaluating the role of the bradykinin receptor antagonist in sepsis using porcine models. The investigators injected *Neisseria meningitidis* in two groups of animals, one of each had placebo and the other pretreated with icatiband. Challenging the previous data, they found no significant differences in outcomes, both groups having massive capillary leakage and cytokines release. Furthermore, bradykinin levels were measured undetectable in both groups [20].

On the other hand, 5 years later, Murugesan and his team demonstrated a significant improvement in outcomes including overall mortality by using a bradykinin-1-receptor antagonist (called BI113823) in rats with polymicrobial severe sepsis (induced by cecal ligation and puncture after sedation). Furthermore, the drug succeeded in mitigating organ injury, having positive effects in respiratory, cardiovascular, and gastrointestinal systems [21].

Even more recently, in 2016, a Spanish paper brought to light the pharmacological difficulties on the development of bradykinin-1-receptor ligand since none of the developed antagonists have optimal pharmacological profile. They built an atomistic model of the receptor aiming to provide deeper insight into its structure, and their experiment allowed to define a common pharmacophore. This finding is potentially useful for the discovery of new compounds [22].

## Discussion

Evidence linking the contact system activation and septic shock is strong in animals, but still not in human models. Furthermore, quantifying its contribution to hypotension and coagulopathy is a difficult task. So far, multiple studies, mostly in non-human models, suggest lethal hypotension are well related to the presence of bradykinin. Coagulopathy, on the other hand, has not been as strongly associated to kallikrein-bradykinin activation and no evidence is available to support this link yet. Being exaggerated vasodilation and hypotension main features of septic shock, potent contributors to capillary leak and multiple organ failure, drugs targeting this system appear to be an attractive option to be better explored. C1 inhibitor concentrates have been used in hereditary angioedema and possess inhibitory properties to the contact system from its initial activation. Such drugs have been only tested for septic shock in a small study, and their effect in mortality and other end points are yet to be determined.

Another group of medications with promising effects are the bradykinin receptor antagonists. Deltiband, a bradykinin-2-receptor, failed to decrease overall mortality, but decreased mortality in the gram-negative sepsis subgroup of patients in one randomized controlled trial. Furthermore, bradykinin-1-receptors have been tested in animal studies with promising results, but so far have not been in human trials on septic shock. Antagonism against both bradykinin receptors is yet to be developed and tested, and a paper was published in 2006 by Morissette and colleagues revealing it is feasible based on a common pharmacophore from existing antagonists [23].

Whether evidence in human subjects is simply lacking or the role of the system in our species is less important remains unknown. Amino acid sequences homology for bradykinin-1-receptors between humans and mouse, rat, rabbit, and dog are, respectively, 68, 71, 78, and 76%. For bradykinin-2-receptors, interspecies homology is greater, being 82, 81, 82.5, and 81% respectively for the above animals. It is therefore recognized there may be a difference of affinity for bradykinin-1-receptor agonists across different species [24], and such differences could explain the results of human clinical trial results. On the other hand, these trials were limited and one of them did show promising results in patients with gram-negative septic shock.

## Conclusions

In summary, although animal based evidence is attractive, many questions remain to be answered regarding this subject. While in multiple studies, the role of contact system seems important, human trials still lack the same confidence. Nevertheless, there is still an open avenue for research to precisely determine the role of contact system activation in human septic shock and also the effect of its inhibition in patients’ outcomes.
